# Opium and Its Votaries

**Published:** 1889-04-06

**Authors:** William Moore

**Affiliations:** K.C.I.E., late Surgeon-General Bombay Staff


### Opium and Its Yotaries.

By Sir William Moore, K.C.I.E., late Surgeon-General Bombay Staff.

Sin Le, a Chinese author, has thus described the effects of opium. 1. It exhausts the animal spirits; hence the youth who smoke will hasten the termination of their years. 2. It wastes the flesh and blood; the faces of the weak who smoke become pale and cadaverous. 3. It dissipates every kind of property. 4. It renders the person ill-favoured; mucus flows from his nostrils and tears from his eyes. 5. It promotes obscenity. 6. It discovers secrets. 7. It violates laws. 8. It attacks vitals. 9. It destroys life. When the opium user has pawned everything in his possession, he will pawn his wife and sell his daughters. Such are the inevitable consequences! European authors have described the opium consumer as betrayed by his "emaciated appearance, lank and shrivelled limbs, tottering gait, sallow visage, feeble voice, and general imbecility." Now there is not the slightest doubt that all Sin Le and others have said about the evil effects of the use of opium, is true as regards many who use the drug immoderately. But it certainly is not fair to lead the public to suppose that the above are the results of the moderate use of opium. At the time that Sin Le wrote as above, the Chinese Government, of whom Sin Le was an official, were fulminating against the introduction of Indian opium into China—posing, indeed, on a high moral standard—while permitting, if not actually encouraging, the extension of opium growing in China. In accordance with the national tendency to overlook one's own failings while condemning the sins of others, we find the daily consumers of alcoholic drinks—swayed by sensational descriptions of opium eaters and opium shops—compounding the sins, they are inclined to by d——g those they have no mind to. Many who would accept the exhibition at a London gin palace or a Calcutta liquor shop as quite a matter of course, are scandalised by the description of opium smoking in the "Mystery of Edwin Drood." There are two sides to most questions, and it is only right and proper that what can be said in favour of opium should be known. Charity, it is said, covers a multitude of sins, and many opium consumers have a right to charitable consideration which is not always accorded. Much ignorance prevails as to the kind of opium which is used. Some swallow little "golis" (petes) of opium, some take "urnall pawnee" (a solution of opium in water), but the majority smoke balls of prepared opium, known as "chandul." Taking crude opium is the most deleterious, and next, taking opium water; for the drug is then brought into immediate contact with the stomach and digestion. But it is questionable if smoking chandul is in any way injurious, and certainly not to the extent it has been asserted to be. Chandul is made by drying, boiling, and straining crude opium; it is not, however, smoked as tobacco is used. A little ball of chandul is put into a peculiarly shaped pipe, which is held in the flame of a lamp. During the preparation of chandul and during the combustion which takes place when it is smoked, much deleterious property is got rid of, and a minimum of narcotic principle is drawn into the mouth with the smoke. For convenience, the chandul smoker reclines, so that the pipe may reach the flame of the lamp which is usually set on a low stool—for poor Easterns do not ordinarily use chairs and tables. Anti-opiumists foolishly make a great point of the fact that opium smokers lie down when they indulge, and John Bull has been called upon to believe in the terrible power of an intoxicating agent which cannot be smoked standing or sitting, as the Briton takes his liquor. But chandul may be smoked standing or sitting, or in any posture. Sir George Birdwood has stated that chandul smoking is a harmless enjoyment, and more recently Dr. Ayres, the colonial surgeon of Hong-Kong, who has paid great attention to the subject, informs us that chandul smoking produces no appreciable effect on the pulse, circulation, or temperature.

Now, people take to the use of opium for many reasons. A very large number do so for the relief of painful maladies, and persons visiting opium shops seeing so many diseased people therein have erroneously concluded that opium causes the ailments to relieve which it is taken. Many in the East commence and continue the use of opium as a prophylactic against the malarious fevers of the country or district in which they reside. Others, of the poorer classes, resort to opium to still the pangs of hunger. Some take opium because they were habituated to it in their childhood, for it is the unfortunate practice of many Eastern women to keep their children quiet with opium, just as in Western countries elixirs and cordials are often used for the same purpose. Another class use opium only when they anticipate a season of great physical fatigue. Others take it simply and entirely for the pleasurable sensations it induces. There is still, however, a class left who resort to it as a solace to the cares and troubles of life, and it is really this class and those suffering from painful ailments who take opium to excess, and who present the characteristic appearances of the confirmed opium consumer.

That opium is the destructive agent it has been portrayed is not in accordance with facts. Many Sikhs take opium habitually, but there are few finer men than the Sikhs. A similar remark applies to the Rajpoots. And the fact that the Chinese penetrate into distant parts of the world, taking their habits and customs with them, and compete successfully with workmen of all other nationalities, sufficiently demonstrates that opium has not yet effected what anti-opiumists anticipate—viz., "the destruction of the Chinese nation!"

Two arguments always brought forward by those condemning opium as "an engine in Satan's hands" are the difficulty of leaving off the habit, and the increasing quantity of the drug required to produce effects. The first assertion is most correct, the latter, say, partially so. Few persons who commence opium even willingly leave off the practice, although it is quite possible for them to do so. But it is only those who take to immoderate smoking who increase the dose. As there are persons who only drink brandy on two occasions, when they have goose for dinner and when they don't, so there are others who never take opium except when they may do so without injury, and when they might not do so. But this is the abuse, not the moderate use of the agent. This is not taking, as St. Paul ordered, a little wine for the stomach's sake. It is rather following the example of the Rev. R. Barham's boon companions, who, their host having been ordered to retire to bed sick, sat drinking till morning. When this good old sort of clerical gentleman came down in the morning, he asked, "John, what time did they go last night?" "Go!" replied John, "go! they are not gone yet: have just rang for a fresh bottle." This, which may have been thought correct in former days, when it was the fashion to get drunk, would now be regarded not only as the abuse of good things, but as something a great deal worse. There is, however, no greater difficulty in refraining from opium than from spirits. There are a very large number of the better classes in China, India, Turkey, and in other countries who only take their opium in moderate quantities. As with spirit drinking so with opium, it is among the lower classes where injurious intemperance prevails. Want, misery, a low moral constitution, absence of education, all tend to the abuse of the one, as of the other.

The quantity taken by the opium consumer depends partly on constitution, or idiosyncrasy, or susceptibility to the action of the drug, and partly on habituation of its use. There is no doubt that many in the habit of using opium consume large quantities. But other powerful or poisonous agents may also be taken in enormous quantities under peculiar idiosyncrasy. Alcohol is a familiar illustration. Arsenic is eaten by the Styrian in proportion sufficient to kill fifty men. Similarly, with opium a tolerance arises from constant use. As an instance of constitutional insensibility towards opium, a case, mentioned by Dr. Christison, may be quoted of a person taking 450 drops of laudanum without any other result than headache and constipation, and whose son at the age of six took sixty minims of muriate of morphia without any apparent result. Confirmed opium eaters have been known to take three ounces a day—in one instance, nine ounces. De Quincy gave a piece of opium to a Malay in the street sufficient to kill some half dozen dragoons together with their horses, and thought the Malay would enjoy himself for six months to come. But the Malay devoured it at once, much to the astonishment and fright of the donor, whose first idea was the forcible exhibition of an emetic. But abuse of the drug cannot be regarded as legitimate use. A consideration of the subject leads to the following conclusions: That opium in moderation, and especially chandul smoking, produces no appreciable ill effects. That the term moderation includes a small amount taken daily, that amount differing according to the peculiar constitution, idiosyncrasy, occupation, habits, and habituation to the drug of the individual. That in many instances and situations the use of opium is actually beneficial—viz., when food is scarce, when exposed in malarious districts, previous to great fatigue, and in many painful maladies. That excessive use of opium is followed by the evils which have been ascribed to it by sensational writers—dyspepsia, premature senility, and decrepitude, and bowel complaint being the chain of evil. That the majority using opium live without any such suffering, sometimes to an advanced age. That a person of ordinary strength of mind may overcome opium craving more readily than drink craving. That the effects of opium are not so injurious as those of alcohol. Many, indeed, have borne witness to this. De Quincy asserted the moderate use of opium to be less injurious than wine. "Wine," he said, "disorders the mental faculties; opium, on the contrary, if taken in a proper manner, introduces among them the most exquisite orders—legislation and harmony." An opium consumer never passes through the different stages of inebriety, the friendly, the argumentative, the captious, the maudlin, the dead drunk. The effects of opium in moderate quantity stop at the first stage of inebriety. No doubt it would be desirable that opium should not be used except medicinally. But the same may be said of spirits. It is a characteristic of the human race to desire some kind of narcotic or stimulant, and notwithstanding all that may be said against it, opium will continue to be used. It is, therefore, better to preach moderation than to fanatically condemn the drug which thousands desire.

